# Factors Predicting the Success of Combined Orbital Decompression and Strabismus Surgery in Thyroid-Associated Orbitopathy

**DOI:** 10.3390/jpm12020186

**Published:** 2022-01-31

**Authors:** Meng-Wei Hsieh, Chih-Kang Hsu, Pao-Cheng Kuo, Hsu-Chieh Chang, Yi-Hao Chen, Ke-Hung Chien

**Affiliations:** 1 Department of Ophthalmology, Taoyuan Armed Forces General Hospital, Taoyuan 325, Taiwan; nsinkoph0311@gmail.com; 2 Department of Ophthalmology, Tri-Service General Hospital Songshan Branch, Songshan, Taipei 105, Taiwan; chikanghsu@gmail.com; 3 Department of Ophthalmology, Tri-Service General Hospital, National Defense Medical Center, Taipei 114, Taiwan; doc30879@mail.ndmctsgh.edu.tw; 4 Department of Orthopedics, Taipei Veterans General Hospital, Taipei 112, Taiwan; king820414@gmail.com; 5 Department of Nursing, Tri-Service General Hospital, National Defense Medical Center, Taipei 114, Taiwan; n3197001@gmail.com; 6 Graduate Institute of Nursing, College of Nursing, Taipei Medical University, Taipei 110, Taiwan

**Keywords:** hyperthyroidism, strabismus, orbital decompression surgery

## Abstract

To evaluate the safety and efficacy of orbital decompression combined with strabismus surgery in thyroid-associated orbitopathy (TAO) and identify factors leading to surgical success. A retrospective comparative case series was conducted on 52 patients who were treated with combined orbital decompression and strabismus surgery. Outcome measurements included perioperative Hertel exophthalmometry and strabismus measurements. Surgical success was defined as binocular single vision (BSV) in the primary and reading positions within 5 prism diopters (PDs). As a result, the average reduction in proptosis was 3.23 mm, with a mean preoperative Hertel measurement of 22.64 mm. Forty-four patients (84.6%) achieved the success criterion and composed the success group. In addition to sex and underlying hyperthyroidism, symmetry of orbitopathy, interocular exophthalmos difference of more than 2 mm, predominant esotropia type, mixed type strabismus, baseline horizontal deviations, baseline vertical deviations, and combination with one-wall decompression surgery were significantly different between the success and failure groups. All complications were mild and temporary. Orbital decompression combined with strabismus surgery produced satisfactory outcomes in selected patients with efficacy and safety. Symmetry between the two eyes with relatively simple strabismus and proptosis ensured surgical success. With experienced surgeons, advanced techniques, and selected patients, this method can serve as an alternative treatment option to minimize the number of surgeries, medical costs and recovery period.

## 1. Introduction

Thyroid-associated orbitopathy (TAO) is a complex disorder associated with autoimmunity against orbital tissues and is characterized by inflammation of retrobulbar tissues, adipogenesis, and the accumulation of glycosaminoglycans within the extraocular muscles [1]. Clinical presentations include proptosis, diplopia, lid retraction, corneal erosion, and visual impairment [2]. The symptoms could be highly variable even between the two eyes of the same patient [3]. Medication is the first line of treatment [4]. However, up to 20% of TAO patients undergo surgical intervention [5].

In 1986, Shorr and Seiff [6] proposed the staged approach in the surgical treatment of TAO patients: (1) orbital decompression, (2) extraocular muscle surgery, (3) eyelid margin repositioning, and (4) blepharoplasty. This approach has become a consensus and has been carried out for most TAO patients requiring surgical intervention. Orbital decompression is often suggested first, as it can cause ocular alignment shifting, affecting strabismic measurements and eyelid positioning [7,8]. Decompression-related disease reactivation is rare but also a concern [9]. Nonetheless, a wide variety of surgical techniques have been introduced and improved over the years, and the rate of patients undergoing orbital decompression for TAO has increased substantially [10]. However, it has been changed since the introduction of Tepezza [11]. Current indications for surgery have also become broad, from cases of optic neuropathy to extreme corneal exposure to cosmetic rejuvenation, and surgical techniques are often individualized for different cases [12,13,14].

The first attempt at simultaneous orbital decompression and eye muscle surgery was described by Michel et al. in 2001 to correct the alignment shifting caused by medial wall decompression. Ocular motility was corrected by medial rectus muscle recession in 58 patients. They achieved free of double vision within margins of 20 degrees in each direction in 31 out of 58 patients after the initial operation [15]. Moreover, studies combining procedures in different stages have emerged in recent years, including orbital decompression surgery combined with eyelid or strabismus surgery. Most studies have shown a reduced number of surgeries without compromising results compared with the staged process [16,17,18,19,20].

In our study, the medical records of 52 TAO patients were reviewed, and all patients underwent combined orbital decompression and strabismus surgery. They were then divided into a success group (44 patients) and a failure group (8 patients). We reviewed the surgical outcomes, the preoperative and postoperative data, and the events of complications and compared the results with those of previous studies. The goal of this study was to evaluate and provide another perspective of the surgical approach to TAO using a combined surgical method for orbital decompression and strabismus correction.

## 2. Materials and Methods

The medical records of patients undergoing combined orbital decompression and strabismus surgery at Tri-Service General Hospital for mild to moderate TAO from January 2015 to December 2020 were retrospectively reviewed. The study was approved by the Institutional Review Board of Tri-Service General Hospital (1-107-05-119).

All patients with hyperthyroidism had received medication treatment at the Endocrinology Department first, and stable disease with euthyroid status was achieved. Data was collected included age, sex, clinical manifestations of TAO, past medical history, and smoking status. Preoperative and postoperative evaluations included visual acuity (VA), intraocular pressure (IOP), a slit lamp exam, Hertel exophthalmometry, extraocular movements (EOMs), and prism tests. A preoperative imaging study was performed for every patient with computed tomography scans (CT) to assess orbital crowding and muscle hypertrophy and aid in surgical planning.

The indications of the combined surgery were as follows: (1) diagnosis of thyroid-associated orbitopathy; (2) disfiguring exophthalmos requiring cosmetic rehabilitation; (3) restrictive strabismus associated with extraocular muscle hypertrophy and symptoms of diplopia; and (4) inactive disease (clinical activity score less than 3) with euthyroid state. Surgery was performed after both euthyroid state after medication control and stable ophthalmic signs with regular follow-ups for more than 6 months were achieved.

All surgeries were performed under general anesthesia by one surgeon (Ke-Hung Chien, MD). A transconjunctival approach was employed for orbital decompression, and adjunctive fat decompression was performed simultaneously if needed. The extent of bone and fat removal was individualized according to the proptosis severity and followed the TAO management protocol [21]. The protocol was mainly determined by Hertel exophthalmeter measurements and the presence of diplopia. Patients were selected for combined surgeries in this study if they had ocular deviation of more than 10 PD in either horizontal or vertical dimension. Strabismus surgery was performed in the same session after orbital decompression, and extraocular muscle recession and/or resection was performed according to preoperative measurements of restrictive strabismus.

Some modifications were made to prevent complications:

(1) We created two separate incisions for different procedures. For example, one inferomedial conjunctival forniceal incision was used for inferomedial orbital decompression, and one inferolateral conjunctival forniceal incision was used for inferior rectus muscle surgery, and inferomedial conjunctival limbal incision was used for medial rectus muscle surgery. Usually, recession of extraocular muscles was done, and resection was performed occasionally to further correct strabismus.
(2) During inferomedial orbital decompression, we performed strut preservation to minimize surgery-induced diplopia and consecutive hypoglobus [22].
(3) During strabismus surgery, we followed the surgical dose response modification with the “intraoperative relaxed muscle positioning technique” [23,24,25,26].

Follow-up was arranged at one week, one month, and three months postoperatively. The clinical outcome measures included Hertel exophthalmometry and strabismus measurements.

The success criteria were set from the literature and modified according to clinical practice [27,28,29]. We defined success in our study as binocular single vision (BSV) in the primary and reading positions within 5 prism diopters (PDs) at least 3 months after the first operation. The criteria of failure were met if the patient had persistent diplopia, had correctable diplopia with more than 5 PDs in the primary and reading positions, or underwent a second operation within 3 months after the first operation.

We used SPSS statistical software version 18 (SPSS Inc, IBM Company, Chicago, IL, USA) to perform statistical analyses. Continuous variables between group 1 and group 2 were analyzed by *t*-test. The variables for the success factors analysis were identified using univariate and multivariate linear regression analyses. A *p* value < 0.05 was considered statistically significant.

## 3. Results

A total of 52 subjects underwent combined orbital decompression and strabismus surgery at Tri-Service General Hospital and were included in the study.

The whole group comprised 35 females and 17 males, with a mean age of 56.07 years ranging from 29.2 to 91.6 years. There were 45 patients who developed TAO with underlying hyperthyroidism, with 3 cases of Hashimoto’s thyroiditis, 2 cases of hypothyroidism, and 2 cases of consecutive hypothyroidism (from thyroidectomy). Mean follow-up time of the patients was 24.7 months, ranging from 3.6 months to 132.7 months (Table 1).

In reviewing the patients’ background prior to the surgery, 24 patients (46.2%) had a history of smoking, including active status and quitting status after TAO diagnosis. All patients had been receiving medical treatment and attained the euthyroid state at least 6 months before surgery, 33 patients (63.5%) had a history of prior usage of corticosteroids, 7 patients (13.5%) underwent radioactive iodine treatment, and 1 patient (1.9%) underwent radiotherapy. The mean duration of TAO was 29.2 months (Table 2).

In the whole group, the patients had a preoperative exophthalmos of 22.64 mm (SD = 3.62) and a postoperative exophthalmos of 19.41 mm (SD = 1.82). Assessing the exophthalmos difference between the two eyes, 47 patients (90.4%) had a relative symmetry of their orbitopathy (interocular exophthalmos difference less than 2 mm). Strabismus exams showed 8 patients (15.4%) with predominant esotropia (with a vertical component less than 5 PDs), 6 patients (11.5%) with predominant hypotropia (with a horizontal component less than 5 PDs), and 38 patients (73.1%) with mixed strabismus. Their mean baseline horizontal deviation was 27.6 PDs (SD = 8.6), and the mean baseline vertical deviation was 9.1 PDs (SD = 6.2). Additionally, 20 patients (38.5%) were found to have cyclotorsion before surgery (Table 3).

Generally, we followed the TAO management protocol [21] with some modifications (detailed in the Methods section). In total, 16 patients (30.8%) underwent single muscle surgery, and 27 patients (51.9%) underwent one-wall decompression in their combination surgery.

To further evaluate the factors in combination surgery, the whole group was then divided into two groups (the success and failure groups) according to their postoperative strabismus status, with a criterion of BSV in the primary and reading positions within 5 PDs at the 3-month follow-up visit. Following the criteria, 44 patients (84.62%) were included in the success group, while 8 (15.38%) were included in the failure group.

Regarding the characteristics of the subgroups, there were significant differences in sex (*p* = 0.04) and underlying hyperthyroidism (*p* = 0.03) (Table 1). In the baseline TAO examinations, symmetry of orbitopathy (*p* = 0.02) and interocular exophthalmos difference more than 2 mm (*p* = 0.03) were significantly different between the groups, while there was no significant difference in other parameters, such as preoperative exophthalmos measurement, smoking status, history of corticosteroid usage, radioactive iodine, radiotherapy, and duration of TAO (Table 2). In the evaluation of operative parameters, predominant esotropia type, mixed-type strabismus, baseline horizontal deviations, baseline vertical deviations, and combination with one-wall decompression surgery were significantly different between the two groups (Table 3).

To determine which parameter most commonly leads to the success of combination surgery, we performed statistical analysis on the relationships among all baseline characteristics and preoperative ophthalmic exams with surgical success. According to the results, symmetry of orbitopathy (*p* < 0.001), interocular exophthalmos difference of more than 2 mm (*p* = 0.012), mixed-type strabismus (*p* = 0.041), baseline vertical deviation (*p* = 0.011) and combination with one-wall decompression surgery (*p* = 0.036) were significantly related to surgical success (all with negative correlations). According to the correlation statistics, interocular exophthalmos difference of greater than 2 mm (r = −0.412), mixed-type strabismus (r = −0.127), and baseline vertical deviation (r = −0.438) had negative correlations, while symmetry of orbitopathy (r = 0.541) and combination with one-wall decompression surgery (r= 0.273) had positive correlations. This result implied that patients with more symmetry between the two orbits and less proptosis more easily tended to experience surgical success in the combination surgery.

There were no major complications noted in our cohort, such as cerebrospinal fluid (CSF) leakage, global injury, or vision loss. However, there were five patients (9.6%) (three patients (6.8%) in the success group and two patients (25%) in the failure group) with temporary infraorbital nerve compromise. One patient in the success group experienced postoperative soft tissue adhesion (symblepharon).

## 4. Discussion

Current minimally invasive surgical decompression techniques have improved surgical outcomes and demonstrated potential in combination with additional procedures [30]. Combination surgeries for TAO management, including two or three of the staged surgeries, are now gaining popularity. In our study, strabismus correction was performed by recessing the hypertrophied extraocular muscles after orbital decompression in the same session. The results showed an 84.6% success rate with the criterion of any postoperative deviations less than 5 PDs. To the best of our knowledge, in addition to our study, there was a study published in 2016 by Seung Woo Choi [18] that reported five patients who underwent combined orbital decompression and strabismus surgery. The study included three cases of lateral wall decompression, one case of balanced decompression, and one case of fat decompression. The mean postoperative Hertel value reduction was 2.1 ± 2.0 mm. The sum of the angles in BSV for the five patients who underwent combined surgery improved from 101.3° to 318.8° (*p* = 0.068). Our study included 52 patients and showed a comparable reduction in exophthalmos measurement (3.23 ± 2.21 mm in the whole group, 3.12 ± 1.73 mm in the success group, and 3.83 ± 3.12 mm in the failure group), even when combined with strabismus surgery. This finding demonstrated that combination surgery could be an effective treatment choice in these patients.

Inferomedial decompression by itself can have widely variable effects, depending on the surgeon technique and extent of bony decompression. For the transnasal and transorbital approaches, studies have shown postoperative diplopia rates ranging from 10% to 35% [31,32,33,34]. The technique was adopted in our study mainly due to surgeon preference and followed the modification to minimize surgically induced diplopia. Modifications of decompression techniques, including inferomedial strut preservation and selective periosteal dissection, have been reported to reduce the incidence of postoperative diplopia [22,35,36,37]. According to our experience, a limited range of decompression with preservation of vital structures can produce a more stable and predictable surgical outcome.

Some studies have reported that lateral wall decompression shows preferable results, with new-onset diplopia rates ranging from 0% to 6% [38,39,40,41,42,43]. This method was adopted in the combined surgery performed in the study of Seung Woo Choi [18], which also produced results comparable to those of staged surgery. However, orbital decompression techniques still lack consistency in outcome, and nonuniform strabismus measurements also limit the ability to make evidence-based decisions [44]. Thus, in regard to practice, surgeon experience and preference still play a major role in adopting decompression techniques. In our study, lateral wall decompression was only considered in patients with a preoperative exophthalmos greater than 24 mm in either balanced decompression or three-wall decompression [21]. The protocol showed reliable results even when combined with strabismus correction in our study.

Studies have reported that 17–45% of TAO patients require more than one procedure to correct TAO-related strabismus [45]. It often causes significant difficulty in daily activities, and early correction may greatly improve quality of life [25,46]. In addition, strabismus surgery is difficult to concur due to significant postoperative drift and different surgical dose responses with common strabismus surgeries [23,24,27,47]. To more precisely evaluate the strabismus result, we modified the intraoperative relaxed muscle positioning technique in which the extent of recession was mainly determined by the position in which the muscle tendon rests freely on the eyeball while in the primary position [25,26]. The technique ensured our surgical expectations during combination surgery and alleviated both the surgeon’s and patient’s burden.

The most alarming complication in the combination surgery is symblepharon from soft tissue adhesion between different surgical planes, which may result in orbital adhesion syndrome. We modified the surgical planes in one session by creating two surgical incisions (one for strabismus surgery and another for orbital decompression) and performing one procedure after closing the other surgical incision. With this method, we encountered only one symblepharon case (1.9%) in the whole group. The symblepharon was relieved by a simple cutting in OPD. There was no abnormality of extraocular movement noted after the symblepharon was relieved.

There were two limitations in our study. First, the study was performed in a retrospective design and lacked a control group. Since staged TAO management is seen as a traditional treatment with proven efficacy, the purpose of our study was to emphasize the relative efficacy of a one-stage operation. We focused on comparing the characteristics between the two subgroups to clarify the factors leading to success. Based on our study results, a further prospective study should be performed to support the benefits of combination surgery. Second, there was only one surgeon involved in the study, and the procedures in the combination surgery were mainly decided by the surgeon’s preference. To minimize the bias of the surgeon’s preference in surgical choice, we followed the TAO management protocol with modifications in the decompression and strabismus techniques. We believe these modifications made our study more practical and reliable in real practice.

## 5. Conclusions

In conclusion, our study shows similar outcomes to those of a previous study performed by Seung Woo Choi [18], which yielded comparable outcomes with the staged process. While there was still a limited number of patients included in our study and more well-designed studies are needed to analyze the safety and effectiveness of the procedure, we believe that with experienced surgeons and advanced techniques, combination surgery can serve as an alternative treatment option to minimize the number of surgeries, medical costs, and recovery period in selected patients.

## Figures and Tables

**Table 1:** Demographic characteristics of the patients in the study.

	Whole Group	Group 1 (Success)	Group 2 (Failure)	*p* Value
No. of subjects (N) (%)	52 (100%)	44 (84.6%)	8 (15.4%)	
Male (N) (%)	17 (32.7%)	15 (34.1%)	2 (25.0%)	0.04 *
Female (N) (%)	35 (67.3%)	29 (65.9%)	6 (75.0%)	
Age at operation (years) (mean) (SD)	56.07 (14.18)	54.95 (13.15)	57.29 (16.38)	0.66
Age at operation (years) (min) (max)	(29.2) (91.6)	(29.2) (77.8)	(36.1) (91.6)	
Follow-up period (month) (mean) (SD)	24.7 (27.9)	28.2 (23.9)	16.44 (8.23)	0.17
Follow-up period (min) (max)	(3.6) (132.7)	(3.6) (132.7)	(4.2) (70.5)	
Underlying hyperthyroidism (N) (%)	45 (86.5%)	39 (88.6%)	6 (75%)	0.03 *

The *p* value in the table is from values compared between Group 1 and Group 2. N = case number. SD = standard deviation. *p* < 0.05 = significant (*).

**Table 2:** Background of patients prior to the surgery.

	Whole Group (N = 52)	Group 1 (Success) (N = 44)	Group 2 (Failure) (N = 8)	*p* Value
Relative symmetry of orbitopathy (Less than 2 mm difference) (N) (%)	47 (90.4%)	42 (93.2%)	5 (62.5%)	0.02 *
Preoperative exophthalmos difference (mm) (mean) (SD)	1.23 (0.86)	1.08 (0.81)	2.05 (0.94)	0.03 *
Preoperative exophthalmos (mm) (mean) (SD)	22.64 (2.56)	22.36 (2.41)	24.18 (4.12)	0.63
Postoperative exophthalmos (mm) (mean) (SD)	19.41 (1.73)	19.24 (1.65)	20.35 (2.13)	0.39
Smoking including active and quit (N) (%)	24 (46.2%)	21 (47.8%)	3 (37.5%)	0.05
Prior corticosteroid usage (N) (%)	33 (63.5%)	28 (63.6%)	5 (62.5%)	0.11
Prior radioactive iodine usage (N) (%)	7 (13.5%)	6 (13.6%)	1 (12.5%)	0.13
Prior radiotherapy (N) (%)	1 (1.9%)	1 (2.3%)	0 (0)	0.06
TAO duration (month) (mean) (SD)	29.27 (13.1)	29.1 (12.3)	30.2 (14.5)	0.15

The *p* value in the table is from values compared between Group 1 and Group 2. N = case number. SD = standard deviation. TAO = thyroid-associated ophthalmopathy. *p* < 0.05 = significant (*).

**Table 3:** Perioperative examinations of the patients in the study.

	Whole Group (N = 52)	Group 1 (Success) (N = 44)	Group 2 (Failure) (N = 8)	*p* Value
Type of strabismus				
Predominantly esotropia (N) (%) (Without other component > 5 PDs)	8 (15.4%)	8 (18.2%)	0 (0)	0.03 *
Predominantly hypotropia (N) (%) (Without other component > 5 PDs)	6 (11.5%)	5 (11.4%)	1 (12.5%)	0.17
Mixed type strabismus (N) (%) (More than one component > 5 PDs)	38 (73.1%)	31 (70.5%)	7 (87.5%)	0.02 *
Baseline horizontal deviations ^1^ (PD) (mean) (SD)	27.6 (8.8)	26.4 (8.5)	34.1 (9.7)	0.03 *
Baseline vertical deviations ^1^ (PD) (mean) (SD)	9.1 (7.0)	8.3 (6.7)	13.6 (7.6)	0.02 *
Cyclotorsion (N) (%)	20 (38.5%)	16 (36.4%)	4 (50%)	0.66
Combination surgery with one muscle (N) (%)	16 (30.8%)	14 (31.8%)	2 (25%)	0.33
Combination surgery with two or more muscles (N) (%)	36 (69.2%)	30 (68.2%)	6 (75%)	0.41
Combination with one-wall decompression (N) (%)	27 (51.9%)	26 (59.1%)	1 (12.5%)	0.02 *
Combination with two or more walls decompression (N) (%)	25 (48.1%)	18 (40.9%)	7 (87.5%)	0.03 *

The *p* value in the table is from values compared between Group 1 and Group 2. N = case number. SD = standard deviation. PD = prism diopter. *p* < 0.05 = significant (*). 1 Deviation of esotropia and hypotropia were in plus numbers while deviation in exotropia and hypertropia were in minus numbers.

## Data Availability

The datasets used and analyzed during the current study are available from the corresponding author on reasonable request.
